# Favipiravir Treatment Prolongs Survival in a Lethal BALB/c Mouse Model of Ebinur Lake Virus Infection

**DOI:** 10.3390/v16040631

**Published:** 2024-04-18

**Authors:** Jingke Geng, Nanjie Ren, Cihan Yang, Fei Wang, Doudou Huang, Sergio Rodriguez, Zhiming Yuan, Han Xia

**Affiliations:** 1 Key Laboratory of Virology and Biosafety, Wuhan Institute of Virology, Chinese Academy of Sciences, Wuhan 430200, China; 2 University of Chinese Academy of Sciences, Beijing 101408, China; 3 Department of Microbiology and Immunology, University of Texas Medical Branch, Galveston, TX 77551, USA; 4 Hubei Jiangxia Laboratory, Wuhan 430207, China

**Keywords:** Ebinur Lake virus, mouse model, favipiravir, ribavirin

## Abstract

*Orthobunyavirus* is the largest and most diverse genus in the family Peribunyaviridae. Orthobunyaviruses are widely distributed globally and pose threats to human and animal health. Ebinur Lake virus (EBIV) is a newly classified Orthobunyavirus detected in China, Russia, and Kenya. This study explored the antiviral effects of two broad-spectrum antiviral drugs, favipiravir and ribavirin, in a BALB/c mouse model. Favipiravir significantly improved the clinical symptoms of infected mice, reduced viral titer and RNA copies in serum, and extended overall survival. The median survival times of mice in the vehicle- and favipiravir-treated groups were 5 and 7 days, respectively. Favipiravir significantly reduced virus titers 10- to 100-fold in sera at all three time points compared to vehicle-treated mice. And favipiravir treatment effectively reduced the virus copies by approximately 10-fold across the three time points, relative to vehicle-treated mice. The findings expand the antiviral spectrum of favipiravir for orthobunyaviruses in vivo.

## 1. Introduction

*Orthobunyavirus* consists of over 120 species that are globally distributed. *Orthobunyavirus* is a genus of the family *Peribunyaviridae*, order *Bunyavirales* [1,2]. Currently, more than 30 orthobunyaviruses are responsible for human and animal diseases, including febrile illness (e.g., Oropouche virus, encephalitis (e.g., La Crosse virus), and hemorrhagic fever (e.g., Ngari virus) [3]. In addition, some orthobunyaviruses, such as Cache Valley virus and Schmallenberg virus, cause abortion and teratogenic effects in ruminants [3,4].

As three-segmented RNA viruses, orthobunyaviruses show high mutation rates and the ability of reassortment, accelerating the evolution and genetic variation of orthobunyaviruses [3,5]. Multiple factors including population growth, deforestation and urbanization, international trade and travel, and climate change have contributed to the expansion of vector active areas and increased vulnerable population to orthobunyaviruses infection [6,7,8,9].

Ebinur Lake virus (EBIV) is a newly classified *Orthobunyavirus* first isolated from pools containing *Culex modestus mosquitos* in Xinjiang province, China [10,11]. Later, EBIV was detected in *Culex* mosquitos, ticks, rats, mongooses, and wild birds collected from Kenya and Russia [12,13]. Our previous work demonstrated that EBIV can infect various cell lines [14], cause acute lethal disease in adult BALB/c mice [15], and spread to *Aedes aegypti mosquito* saliva glands upon blood feeding [16]. These findings indicate that EBIV may have a wide distribution and potential for zoonotic transmission from animal to human.

Favipiravir (T-705) is a broad-spectrum inhibitor for RNA viruses, with good efficacy in a variety of bunyaviruses. It inhibits Crimean–Congo hemorrhagic fever [17,18,19], Rift Valley fever virus [18,20,21], Punta Toro virus [18,22], and severe fever with thrombocytopenia virus [18,23] in vivo.

Ribavirin is a guanosine analog and a Food and Drug Administration (FDA)-approved drug with broad-spectrum activity against RNA viruses [21,24]. Ribavirin has exhibited efficacy at inhibiting a variety of bunyaviruses in vivo, such as Crimean–Congo hemorrhagic fever [19], Rift Valley fever virus and Punta Toro virus [21,25], and severe fever with thrombocytopenia virus [26].

Our previous studies found that ribavirin had a dose-dependent inhibitory effect on EBIV in BHK-21 cells, but favipiravir was ineffective, even at 100 μM [27]. To investigate whether favipiravir and ribavirin can antagonize EBIV in vivo, we evaluated the antiviral activity of these two compounds in a mouse model of lethal EBIV infection.

## 2. Materials and Methods

### 2.1. Ethics Statement

Animal experiments were approved by the Laboratory Animal Ethics Committee of Wuhan Institute of Virology, Chinese Academy of Sciences, Wuhan, China (permit number: WIVA12202301). All animal procedures were performed in accordance with the ethical guidelines.

### 2.2. Animals

Adult BALB/c female mice (6–8 weeks of age) were provided by the Animal Centre of Wuhan Institute of Virology. The mice were maintained in the ABSL-2 facility with controlled temperature (22 °C) and humidity and a 12 h light/dark cycle.

### 2.3. Virus

EBIV isolate Cu-XJ20 was first isolated from *Culex modestus mosquitos* in Xinjiang, China, as previously described [14]. The EBIV working stock was propagated in BHK-21 cells in Dulbecco’s modified Eagle’s medium (DMEM) containing 2% fetal bovine serum, (FBS), aliquoted and stored at −80°C. The infectious dose was determined via a plaque assay as previously described [14]. The results are expressed as plaque forming units (PFU) mL^−1^. All in vitro work with infectious virus was performed in a biosafety level-2 (BSL-2) laboratory.

### 2.4. Antiviral Compounds

Favipiravir and ribavirin were purchased from MedChemExpress (New Jersey, USA). Both compounds were dissolved in 2.9% sodium bicarbonate (5.6% sodium bicarbonate solution diluted with sterile water; the pH is approximately 7.5) for administration via intraperitoneal (i.p.) injection.

### 2.5. Evaluation of In Vivo Antiviral Efficacy

The lethal dose of EBIV for BALB/c mice was determined previously with an LD_50_ of 0.046 PFU [15]. We used 10 PFUs (~200 LD_50_ per mouse) as the infectious dose in the following two independent experiments.

#### 2.5.1. Experiment 1

Mice in each group (n = 9 per group) were inoculated i.p. with 100 µL serum-free DMEM containing 10 PFU EBIV. Favipiravir 300 mg/kg/day or ribavirin 100 mg/kg/day were i.p. injected two days prior to EBIV infection and administered every 12 h. The mock infection control group (negative control) received an equal volume of vehicle (2.9% carbonic acid-sodium bicarbonate) and is hereafter referred to as the vehicle-treated group. Healthy controls (n = 9) were inoculated i.p. with 100 µL serum-free DMEM and received an i.p. injection of an equal volume of vehicle (2.9% sodium bicarbonate). The treatment was continued until death or 25% decline in body weight.

The signs of disease (piloerection, hunched posture, lethargy, body tremble, immobile, and weak/labored breathing), survival, and body weight of mice in each group were monitored daily. Viral titers and viral RNA copies on 1, 3, and 5 days post-injection (dpi) in the serum were determined using a plaque assay and qRT-PCR, respectively. Complete blood counts were also performed.

#### 2.5.2. Experiment 2

Mice in each group (n = 15 per group) were inoculated i.p. with 100 µL serum-free DMEM containing 10 PFU EBIV. Administration of favipiravir (i.p.) was started two days before infection and administered every 12 h at a dosage of 300 mg/kg/day. The mock infection control group (negative control) received an equal volume of vehicle (2.9% carbonic acid-sodium bicarbonate) and is hereafter referred to as the vehicle-treated group. The treatment continued until 5 dpi.

The signs of disease (piloerection, hunched posture, lethargy, body tremble, immobile, and weak/labored breathing) and body weight of mice in each group were monitored daily. Five mice in each group were randomly sacrificed at 1, 3, and 5 dpi, respectively. Five healthy mice were also sacrificed to serve as healthy controls. Gross examination was performed, and tissues were collected for hematoxylin and eosin (HE) staining to analyze tissue lesions. Before sacrifice, systemic blood was collected through a terminal retroorbital bleed for biochemical, cytokine, and viral load analyses.

### 2.6. Virus Titration of Serum

To determine the virus titer in serum, blood samples were kept at 4 °C and centrifugated at 3000× *g* for 20 min to separate the serum. Sera were stored at −80 °C until experimental use. Virus titers were determined using a plaque assay as previously described [14]. The results are expressed as PFU mL^−1^.

### 2.7. Quantitative Reverse Transcription Real-Time PCR (qRT-PCR)

RNA was extracted using an automated nucleic acid extraction system following the manufacturer’s instructions (NanoMagBio; Wuhan, China). The qRT-PCR was performed using the CFX96 Touch Real-Time PCR Detection System (Bio-Rad; Hercules, CA, USA) and HiScript II U+ One Step qRT-PCR Probe Kit (Vazyme; Nanjing, China). The equation for the standard curve was as follows:

y = −4.8x + 67.76

where x denotes lg (copies/mL of EBIV), y denotes the Ct value, and the R^2^ is 0.9997. The curve was generated using 10-fold serial dilutions of EBIV RNA and used to calculate the EBIV RNA load in each sample. The primers used have been previously described [14]. The primers and probes targeting the S segment were as follows: probe (5′-FAM-TTTTGGGTCCATCTCTTTCCTCTGC-TAMRA-3′) and primers (forward: 5′-GGTACCTCTGGCGCATTGTCTTTTC-3′; reverse: 5′-GAAAAATGGCATCACCTGGGAAAGT-3′).

### 2.8. Hematology and Blood Chemistry

All cell counts were quantified using the HemaVet 950FS Hematology Analyzer (DREW; Dallas, TX, USA). Measurements included counts of white blood cells, red blood cells, hemoglobin concentration, platelets, neutrophils, lymphocytes, monocytes, eosinophils, and basophils.

Blood biochemistry was performed to analyze the levels of alanine transaminase, creatine kinase, and lactate dehydrogenase-L (LDH-L) using a Chemary 800 automatic biochemical analyzer. The assays were performed by Wuhan Servicebio Technology Co., Ltd. (Wuhan, China).

### 2.9. Cytokine Assay

The levels of cytokines in serum were determined using a Luminex cytokine analysis (RnD), including tumor necrosis factor-alpha (TNF-α), interferon-gamma (INF-γ), interleukin (IL)-10, IL-2, IL-4, and IL-5. The determinations were made by Shanghai LabEx Biotech Co., Ltd. (Shanghai, China).

### 2.10. Histopathology Assay

For the histopathological analysis, tissues of mice in the favipiravir-infected and vehicle-treated group fixed with 4% paraformaldehyde were embedded in paraffin, sectioned, stained with HE, and examined using light microscopy. The assays were performed by Wuhan Servicebio Technology Co., Ltd. (Wuhan, China).

### 2.11. Statistical Analyses

The Mantel–Cox log-rank test was used to analyze Kaplan–Meier survival. Hematology and blood chemistry analyzes were performed using Student’s two-tailed *t*-test. Viral titers were analyzed using the Mann-Whitney U test. All statistical evaluations were performed using Prism software (version 9, GraphPad Software).

## 3. Results

### 3.1. Favipiravir Prolongs Survival Time and Reduces Viral Load for EBIV-Infected Mice

**Figure 1:**
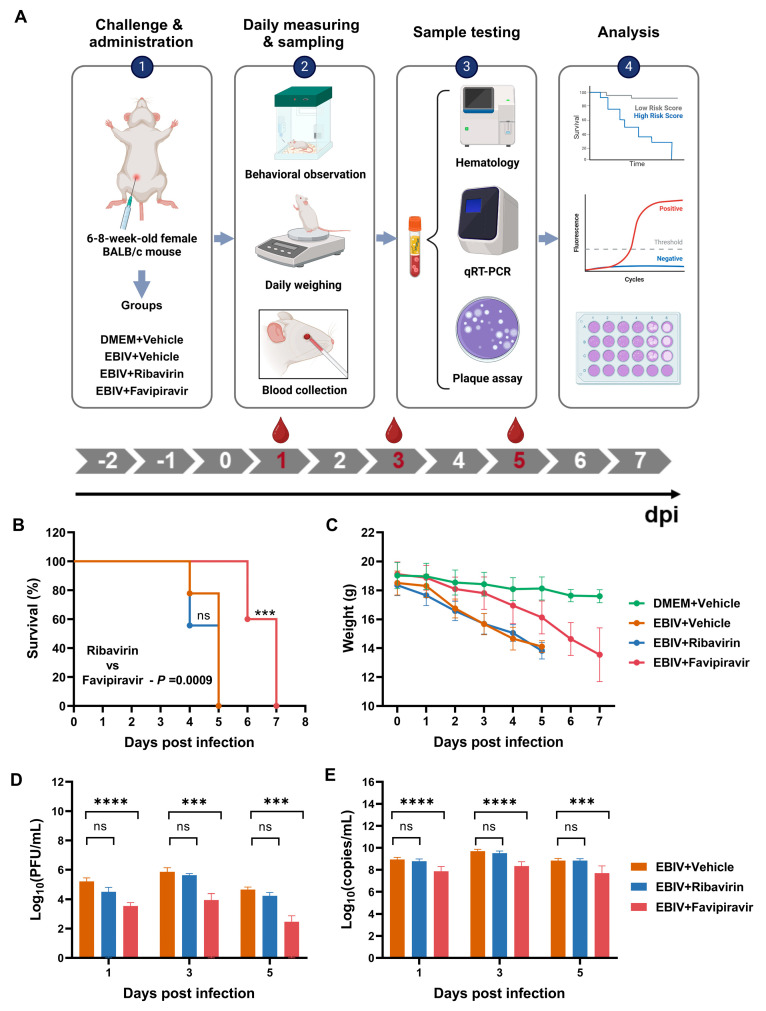
Experimental strategy, survival curve, body weight changes, and viral load in EBIV-infected BALB/c mice. Nine mice in each group were inoculated intraperitoneally with EBIV or DMEM as a control, and treated with vehicle, favipiravir, or ribavirin. The dose of favipiravir was 300 mg/kg/day and the dose of ribavirin was 100 mg/kg/day, which is the highest dose most often used in multiple publications [19,25,28]. The treatment (administrated every 12 h) began at 2 days prior to EBIV infection and continued until death or 25% decline in body weight for the mice (**A**) Experimental design. (**B**) Survival rate determined using the Kaplan-Meier analysis. (**C**) Body weight data expressed as the group mean and standard error. (**D**,**E**) Virus titers and RNA copies on 1, 3, and 5 dpi in sera determined using plaque assay (**D**) and qRT-PCR (**E**), respectively. Asterisks denote significant differences (*** *p* < 0.001, **** *p* < 0.0001, ns: no significant difference).

We evaluated whether favipiravir, or ribavirin could protect BALB/c mice from EBIV infection (Figure 1A). The curves of survival and body weight changes in EBIV-infected BALB/c mice treated with vehicle, favipiravir, and ribavirin are shown in Figure 1B,C.

No animal survived after favipiravir or ribavirin treatment. The median survival times of mice in the vehicle-, favipiravir-, and ribavirin-treated groups were 5, 7, and 5 days, respectively (Figure 1B). Mice in the vehicle-treated group began to die at 4 dpi, and the body weight of all mice dropped by more than 25% on 5 dpi, meeting the criteria for euthanasia (Figure 1B,C). Compared with the vehicle-treated group, favipiravir treatment significantly prolonged the survival time of mice (*p* = 0.0004). Mice in the favipiravir-treated group began to die at 6 dpi, and all mice had died or lost more than 25% of their weight at 7 dpi (Figure 1B). Consistent with the vehicle-treated group, ribavirin-treated mice showed almost the same survival curves without significant differences (Figure 1B).

Body weight changes are also an important indicator for evaluating the antiviral effects of ribavirin and favipiravir. The body weight of the mice in the vehicle-treated group dropped sharply at 2 dpi and continued to decrease throughout the infection (Figure 1C). Favipiravir treatment slowed the weight loss trend of mice. Within 3 dpi, the weight of mice in the favipiravir-treated group was not significantly different from that of healthy control mice (Figure 1C). The onset of clinical symptoms of mice in the vehicle-treated group appeared from 2 dpi and included piloerection, hunched posture, lethargy, body tremble, immobile, and weak/labored breathing. However, favipiravir-treated mice were visibly healthy, and the onset of clinical symptoms (piloerection) was delayed until 5 dpi. Other clinical symptoms occurred between 6 and 7 dpi and included hunched posture, lethargy, body tremble, immobile, and weak/labored breathing. Consistent with the vehicle-treated group, mice in the ribavirin-treated group had almost the same body weight change curves and clinical symptoms without significant differences (Figure 1C).

To evaluate the effect of ribavirin and favipiravir treatments on viral load in sera of EBIV-infected mice, blood samples were collected on 1, 3, and 5 dpi from each group (Figure 1A). To monitor the virus titer, we directly detected live virus titers in the serum at three time points using plaque assay. Favipiravir significantly reduced virus titers 10- to 100-fold in sera at all three time points compared to vehicle-treated mice (Figure 1D). However, ribavirin failed to reduce viral titers in mouse serum (Figure 1D). To detect viral RNA copies, RNA was extracted from sera and the S segment RNA of EBIV was detected by qRT-PCR. As shown in Figure 1E, across the three time points, favipiravir treatment effectively reduced the virus copies by approximately 10-fold, relative to vehicle-treated mice (Figure 1E). However, no significant difference was detected between ribavirin- and vehicle-treated groups at all time points (Figure 1E).

### 3.2. Hematology Analyses of Untreated and Treated EBIV-Infected Mice

Blood samples collected on 1, 3, and 5 dpi were analyzed for complete blood counts. To evaluate whether ribavirin or favipiravir treatment can restore blood cell counts to healthy levels in mice or alleviate abnormal blood cell counts in mice relative to the vehicle-treated group mice, we compared the differences in blood cell counts of mice in the healthy control, vehicle-treated, favipiravir-treated, and ribavirin-treated groups. Statistical differences were calculated in vehicle-treated vs. healthy control, favipiravir-treated vs. vehicle-treated, favipiravir-treated vs. healthy control, ribavirin-treated vs. vehicle-treated, and ribavirin-treated vs. healthy control.

**Figure 2:**
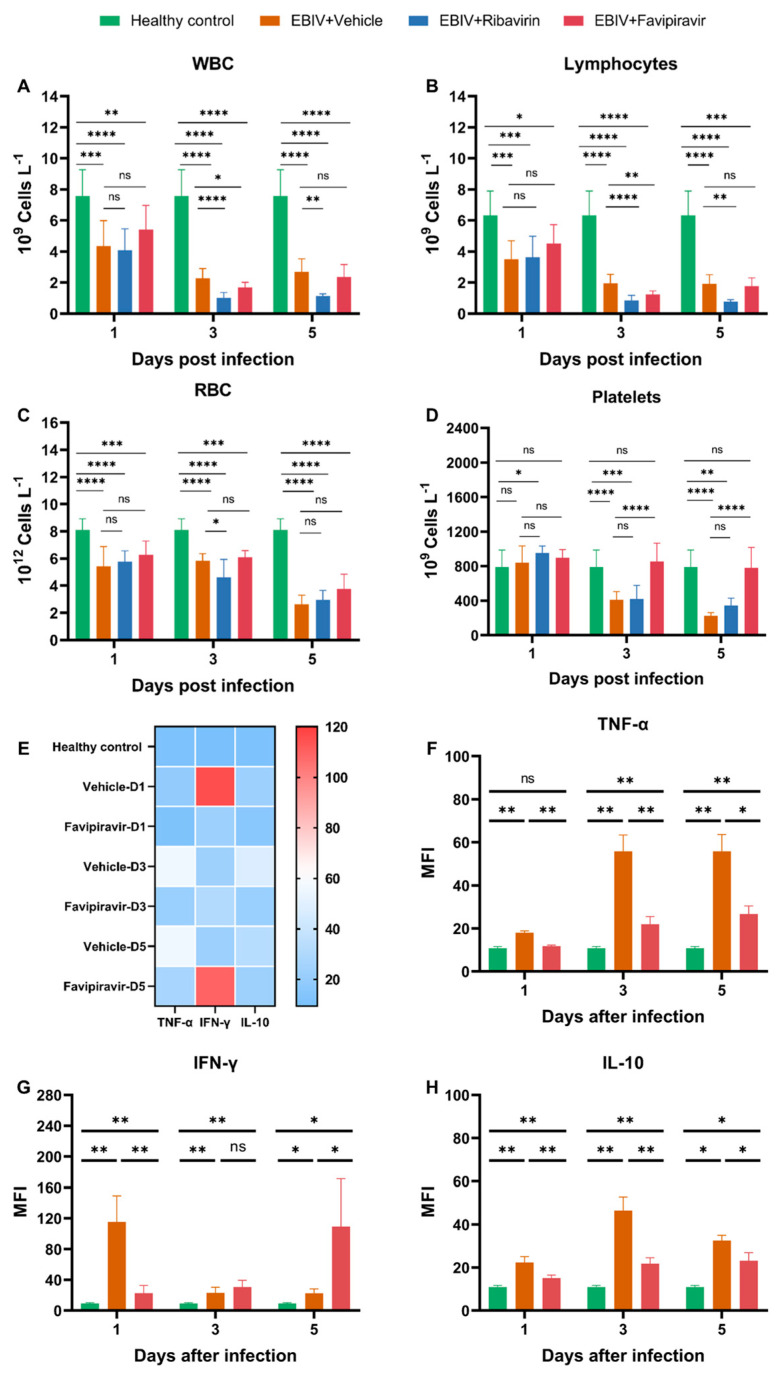
Hematologic and cytokine analyses on 1, 3, and 5 dpi in untreated and treated EBIV-infected mice. The count of (**A**) total white blood cells (WBCs), (**B**) lymphocytes, (**C**) total red blood cells (RBC), and (**D**) platelets. (**E**) Heatmap analysis of cytokine. Level of (**F**) TNF-α, (**G**) IFN-γ, and (**H**) IL-10. Healthy control were the mice without infection; mice in infection groups were inoculated intraperitoneally with 10 PFU EBIV and treated intraperitoneally with vehicle, favipiravir, or ribavirin. The treatment (administrated every 12 h) began at 2 days prior to virus infection and continued until death or 25% decline in body weight for the mice. * *p* < 0.05, ** *p* < 0.01, *** *p* < 0.001, **** *p* < 0.0001, ns: no significant difference.

The total number of white blood cells (Figure 2A), neutrophils (Figure S2A), and lymphocytes (Figure 2B) in the vehicle-treated mice decreased significantly at 1, 3, and 5 dpi. Red blood cells (Figure 2C) and hemoglobin (Figure S2A) of mice in the vehicle-treated group decreased slightly at 1 and 3 dpi and decreased significantly at 5 dpi. Platelets did not decreased significantly at 1 dpi and decreased significantly at 3 and 5 dpi (Figure 2D).

Treatment with favipiravir prevented the reduction of platelets (Figure 2D). Starting from 1 dpi, the platelets in the favipiravir-treated mice were not significantly different from that in healthy control group. Starting from 3 dpi, there was a significant difference between the platelets in the favipiravir- and vehicle-treated groups (Figure 2D). However, favipiravir did not improve the counts of other blood cells in mice, including total white blood cells, lymphocytes, red blood cells, neutrophils, and hemoglobin (Figure 2A–C and Figure S2A). At 3 dpi, mice in the favipiravir-treated group had lower white blood cells, lymphocytes and neutrophils than those in the vehicle-treated group, and had lower neutrophils than those in the vehicle-treated group at 5 dpi. Eosinophils in the vehicle-treated mice increased at 5 dpi, and favipiravir treatment restored these counts to healthy levels (Figure S2A). Basophils in the vehicle-treated mice decreased at 3 dpi, but increased at 5 dpi. There were no significant differences in basophils between mice in favipiravir-treated and healthy control groups (Figure S2A). There were no significant differences in monocytes between mice in favipiravir-treated and healthy control groups (Figure S2A).

Ribavirin treatment did not restore blood cell counts of mice to healthy levels (Figure 2A–D and Figure S2A). Moreover, some blood cell counts in the ribavirin-treated group were lower than those in the vehicle-treated group at some time points, such as white blood cells, lymphocytes, red blood cells, neutrophils, monocytes, and hemoglobin (Figure 2A–C and Figure S2A). There were no significant differences in eosinophils and basophils between mice in ribavirin- and vehicle-treated groups (Figure S2A).

### 3.3. Cytokine Analyses of Untreated and Treated EBIV-Infected Mice

Blood samples collected on 1, 3, and 5 dpi were analyzed for cytokine levels. To evaluate whether favipiravir treatment can restore cytokine levels to healthy levels in mice or alleviate inflammation in mice relative to the vehicle-treated group mice, we compared the differences in cytokine levels in the healthy control group, vehicle-treated group, and favipiravir-treated group. Statistical differences were calculated in favipiravir-treated vs. vehicle-treated, favipiravir-treated vs. healthy control, and vehicle-treated vs. healthy control groups.

The TNF-α and IL-10 levels of mice in the vehicle-treated group began to increase from 1 dpi and were significantly different from those in the healthy control group. Although treatment with favipiravir did not restore TNF-α and IL-10 levels to healthy levels, the treatment significantly reduced TNF-α and IL-10 levels in serum (Figure 2E,F,H).

IFN-γ levels of mice in the vehicle-treated group increased sharply at 1 dpi and decreased at 3 and 5 dpi but were always significantly different from those in the healthy group (Figure 2E,G). Favipiravir treatment significantly reduced the IFN-γ levels in sera at 1 dpi (Figure 2E,G). At 3 dpi, there was no significant difference in IFN-γ levels between the favipiravir- and vehicle-treated groups (Figure 2E,G). However, at 5 dpi, the IFN-γ level in the favipiravir-treated group was higher than that of the vehicle-treated group (Figure 2E,G). Favipiravir was unable to restore IFN-γ levels to healthy levels throughout treatment (Figure 2E,G).

No significant changes in IL-2, IL-4, and IL-5 were evident in the healthy control, vehicle-treated, and favipiravir-treated groups (Figure S2B,C).

### 3.4. Gross Pathology of Untreated and Treated EBIV-Infected Mice

**Figure 3:**
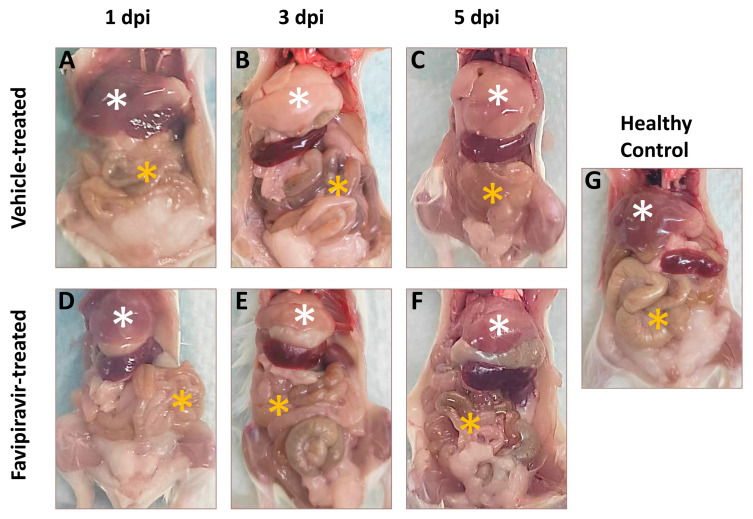
Gross pathology of mice in the healthy, favipiravir-treated, and vehicle-treated groups at 1, 3, and 5 days post infection. Fifteen mice in each group were infected intraperitoneally with 10 PFU EBIV and treated intraperitoneally with favipiravir or vehicle. The treatment (administrated every 12 h) began 2 days prior to EBIV infection and continued until 5 dpi. Five mice in each group were randomly sacrificed at 1, 3, and 5 dpi. Five healthy mice were also sacrificed to serve as healthy controls. (**A**–**C**) represent gross pathology of mice in the vehicle-treated group at 1, 3, and 5 dpi of EBIV infection, respectively. (**D**–**F**) represent gross pathology of mice in the Favipiravir-treated group at 1, 3, and 5 dpi of EBIV infection, respectively. (**G**) represents the gross pathology of healthy mice. The white stars indicate the mouse’s liver and the orange stars indicate the mouse’s intestine.

After infection with EBIV, significant pathological changes in peripheral organs such as the liver and intestine were evident in BALB/c mice. Compared with the healthy control, no obvious lesions were visible in liver and intestine samples, with no significant differences from the healthy control at 1 dpi (Figure 3A,D,G, white star and orange star). At 3 and 5 dpi, the color of the liver tissue in the vehicle-treated group was significantly lighter (Figure 3B,C, white star). At 3 dpi, the intestine in the vehicle-treated group began to show devoid of contents and accompanied by severe congestion (Figure 3B, orange star), and severe deformation occured at 5 dpi. After treatment with favipiravir, the liver color was not light and there was no further congestion in the intestines (Figure 3E,F, white star and orange star).

### 3.5. Histopathological Changes in Untreated and Treated EBIV-Infected Mice

To evaluate whether favipiravir reduces the extent of lesions in EBIV-infected mice, selected tissues that from the spleen, intestine, brain, and liver were collected and processed for HE staining.

**Figure 4:**
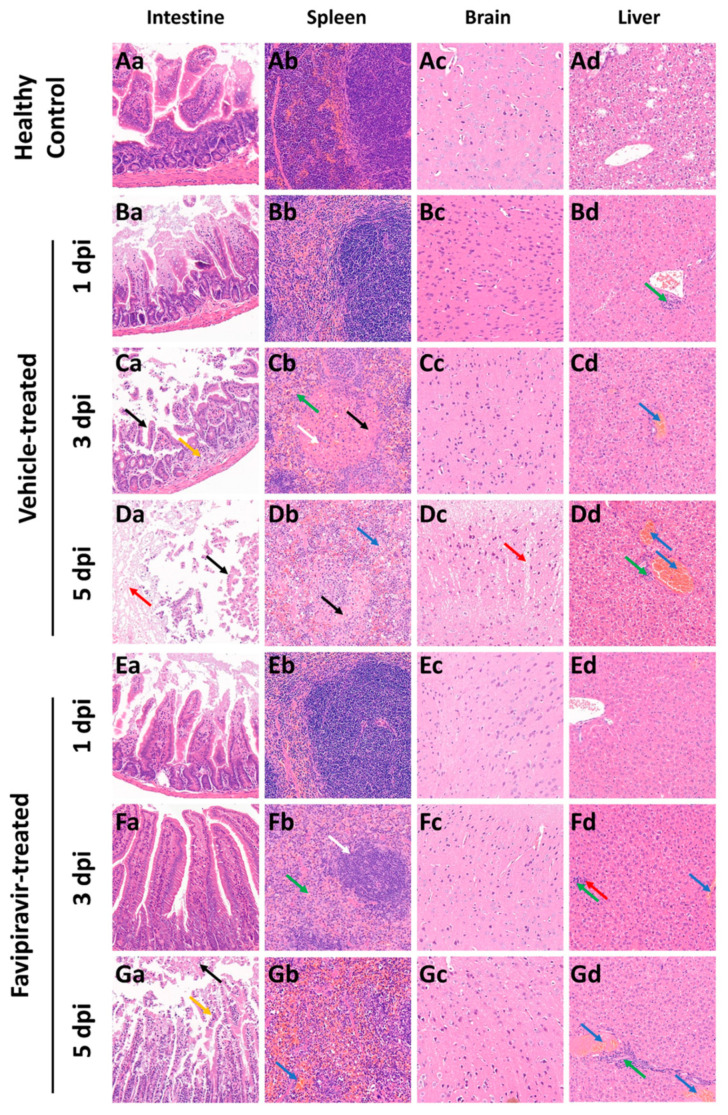
Histopathological changes in tissues from healthy and EBIV-infected BALB/c mice. HE staining revealed pathological lesions in sections of the liver, intestine, brain, and spleen of BALB/c mice treated with vehicle or favipiravir and infected with EBIV. Fifteen mice in each group were infected intraperitoneally with 10 PFU EBIV and treated intraperitoneally with favipiravir or vehicle. The treatment (administrated every 12 h) began 2 days prior to virus infection and continued until 5 dpi. Tissues were harvested on 1, 3, and 5 dpi from mice of the vehicle-and favipiravir-treated groups. Healthy mice were also sacrificed to serve as healthy controls. Tissue damage was identified and is indicated by arrows (magnification, 200×). (**Aa**–**Ad**) Intestines, spleen, brain and liver of the healthy mice, respectively. (**Ba**–**Bd**) Intestines, spleens, brains, and livers of vehicle-treated group of mice at 1 dpi of EBIV infection, respectively. (**Ca**–**Cd**) Intestines, spleens, brains and livers of mice in the vehicle-treated group at 3 dpi of EBIV infection, respectively. (**Da**–**Dd**) Intestines, spleens, brains and livers of mice in the vehicle-treated group at 5 dpi of EBIV infection, respectively. (**Ea**–**Ed**) Intestines, spleens, brains and livers of mice in the Favipiravir-treated group at 1 dpi of EBIV infection, respectively. (**Fa**–**Fd**) Intestines, spleens, brains and livers of Favipiravir-treated group mice at 3 dpi of EBIV infection, respectively. (**Ga**–**Gd**) Intestines, spleens, brains and livers of Favipiravir-treated group mice at 5 dpi of EBIV infection, respectively. The lesions indicated by the different arrows are explained in the text.

Similar to the results of gross examination, favipiravir treatment alleviated the intestinal lesions in mice. At 1 dpi, there were no obvious abnormalities in the intestines of mice in the vehicle- and favipiravir-treated groups compared to the healthy control (Figure 4Ba,Ea). At 3 dpi, in the vehicle-treated group, the intestine of mice showed a mild reduction in intestinal glands and a small amount of hyperplasia of connective tissue in the lamina propria (Figure 4Ca, orange arrow) as well as the shedding of a large number of epithelial cells (Figure 4Ca, black arrow). At 5 dpi, many epithelial cells were shed in the intestinal tissue (Figure 4Da, black arrow), a large area of mucosal layer was autolyzed (Figure 4Da, red arrow), and the structure was blurry. In the intestine of the mice in the favipiravir-treated group, at 3 dpi, the intestinal glands in the lamina propria were abundant, and the muscle fiber structure was normal and arranged regularly. At 5 dpi, many epithelial cells were shed in the mucosal layer (Figure 4Ga, black arrow) and epithelial cell necrosis was rare (Figure 4Ga, orange arrow). However, intestinal glands in the lamina propria were abundant and the muscle fiber structure was normal and arranged regularly.

Favipiravir treatment could alleviate spleen lesions in mice. At 1 dpi, the spleens of mice in the vehicle-and favipiravir-treated groups displayed a clear structure and no obvious abnormalities (Figure 4Bb,Eb). At 3 and 5 dpi, the boundary between red and white pulp of mice in the vehicle-treated group was blurry, splenic nodules were small (Figure 4Cb, white arrow), and parenchymal cells were necrotic (Figure 4Cb,Db, black arrow). At 3 dpi, granulocytic infiltration was seen but was rare (Figure 4Cb, green arrow). At 5 dpi, extramedullary hematopoietic cells were visible (Figure 4Db, blue arrow). In the favipiravir-treated group, at 3 dpi, the boundary between red pulp and white pulp was clear, but the splenic nodules in the spleen were small in size (Figure 4Fb, white arrow) and a small amount of granulocyte infiltration was evident in the red pulp (Figure 4Fb, green arrow). At 5 dpi, the boundary of red pulp and white pulp was clear, the structure of each layer of the white pulp was clear, and there was no obvious change in number and size. However, mild congestion was seen in the red pulp (Figure 4Gb, blue arrow).

The cortexes of mice in the vehicle-treated group had loose structures (Figure 4Dc, red arrow) in many places at 5 dpi. No obvious abnormalities were found in the brain tissue of mice in the favipiravir-treated group.

Treatment with favipiravir did not reduce the degree of lesions in the liver. At 1 dpi, in the livers of mice in the vehicle-treated group, a small amount of lymphocyte infiltration could be seen around the bile ducts in the local portal area. At 3 dpi, venous congestion was evident (Figure 4Cd,Fd, blue arrow) in the livers of mice in the vehicle-and favipiravir-treated groups; mice in the favipiravir-treated group displayed punctate lymphocyte infiltration (Figure 4Fd, green arrow) and rare punctate necrosis of liver cells (Figure 4Fd, red arrow). At 5 dpi, the mice in the vehicle-and favipiravir-treated groups had more venous congestion in the liver (Figure 4Dd,Gd, blue arrow), and a small amount of lymphocyte infiltration could be seen around the bile ducts in the local portal area (Figure 4Dd,Gd, green arrow).

## 4. Discussion

To our knowledge, the immunocompetent animals infection model was only successfully developed for three orthobunyaviruses: Jamestown Canyon virus [28], Cache Valley virus [29,30] and Bunyamwera virus [30]. Most animal infection model were based on immunodeficient animals (e.g., interferon knockout) or immunologically immature animals (e.g., newborn, suckling, or weanling) [30], since the orthobunyaviruses they used do not cause disease in immunocompetent laboratory animals [31]. When studying pathogenesis in immunocompetent individuals, the immune system often plays a key role and may particularly influence virus infection or contributing to immunopathology, and it represents a more realistic and predictive context in which antiviral therapies can be tested [30]. To address the needs to better understand the pathology and experimental countermeasures for newly classified orthobunyaviruses, from the context of an intact immune system, we developed a lethal infectious murine model for EBIV in immunocompetent BALB/c mice [15].

Favipiravir exhibits broad in vitro and in vivo activities against many RNA viruses. In this study, the survival rate of mice in the favipiravir-treated group did not improve, but the survival time of the mice was extended by 2 days. At the same time, the viral load in sera was significantly reduced, the weight loss trended slower, and the onset of clinical symptoms was delayed. Blood biochemistry (Figure S3) and HE staining results show that treatment with favipiravir cannot alleviate some tissues lesions in mice; the mice eventually succumbed to EBIV infection. This was likely due to the sensitivities of BALB/c mice to EBIV. Thus, favipiravir was unable to provide complete protection. Ribavirin did not prolong the survival time of mice, and the mice developed clinical symptoms on the second day after EBIV infection, with continued decreasing body weight throughout the infection process, consistent with the vehicle-treated group. This result is similar to the report of Jamestown Canyon virus, where favipiravir only extended the survival time of mice, while ribavirin had no effect in a lethal C57BL/6J mouse model [28].

In previous studies, we found that favipiravir showed no antiviral activity against EBIV in BHK-21 cells within 100 μM [27], but the protective effect could be observed for favipiravir in vivo here. This may be because favipiravir is a prodrug, which needs to convert to its active ribonucleoside 5′-triphosphate by intracellular host kinases, then acts as a nucleotide analog to selectively inhibit RNA-dependent RNA polymerase (RdRp) and induce lethal mutagenesis [25,32,33]. And the conversion rate for this process likely varies from cell line to cell line [25]. The concentration of favipiravir used in our previous study may be not sufficient to show antiviral effects due to the low conversion rate.

Favipiravir was approved in Japan for the treatment of influenza in 2014 [32,33,34]. As a broad-spectrum antiviral compound, favipiravir has demonstrated antiviral effects against a wide range of viruses in a variety of animal models. In the hamster model, favipiravir could reduce the SARS-CoV-2 infectious titers in lungs and clinically alleviate histopathological damage in the lungs [32]. Favipiravir could protect against lethal CCHFV challenge in IF-NAR(−/−) mice and prevent significant weight loss, and it also protected 100% of MA-CCHFV infected WT-C57BL6/J mice [19]. In cynomolgus macaques, favipiravir treatment was effective in reducing viral load for ZIKV in plasma [34]. In the guinea pig model, favipiravir could reduce the infectious LASV titers in the serum, liver, spleen and lungs by 2−3 logs compared with the placebo control group [35,36]. 

Five primary distinct mechanisms have been proposed to explain the antiviral properties of ribavirin: competitive inhibition of inosine monophosphate dehydrogenase (IMPDH) by RMP resulting in the depletion of guanine nucleotides, impact on host cell immunity, inhibition of mRNA capping, inhibition of viral RNA-dependent RNA-polymerase (RdRp), and enhancement of viral mutagenesis by substitution of RTP for GTP [19,37,38]. It has been reported that Ribavirin can effectively inhibit Jamestown Canyon virus (JCV) and La Cross virus (LACV) infection in vitro but does not exhibit antiviral activity in the lethal C57BL/6J mouse model of JCV infection or Swiss Webster female mice of LACV infection [28,39]. This is similar to our findings that ribavirin could inhibit EBIV in vitro [27] but had no antiviral activity in vivo. But the exact reasons that ribavirin cannot work efficiency in vivo is still unknown and needs to be further explored.

There are several limitations to this study. Firstly, the antiviral efficacy may be related to strain and gender of animal models. For example, ribavirin is effective against Crimean-Congo hemorrhagic fever virus in STAT1(−/−) mice but fails to provide protection in IFNAR(−/−) mice [19]. Animals exhibit sex-based differences in immune response. Additionally, hormonal differences between male and female mice can affect the progression and severity of viral infections. For example, the survival rates of female and male mice were inconsistent when favipiravir was used to treat ZIKV in the IFNAR(−/−) mice model [40]. Here, we only tested one animal model, adult female BALB/c mice. Secondly, the administration method used may not maintain high concentrations of the compound in the mice for a long time, which will influence the antiviral efficiency in vivo. Pharmacokinetic data of favipiravir in mice indicate that only 10% of the drug remains 6 h following a single per os administration [25]. Based on pharmacokinetics, injection every 6 h is ideal, but multiple injections may further burden the mice. A reduction in intracellular GTP levels by ribavirin can lead to hemolytic anemia, which may interfere with further use of ribavirin [24]. There may be a synergistic effect when ribavirin is used together with favipiravir, which allows ribavirin to continue to work [24]. However, in this study, we only tested a single dose and a single treatment.

In summary, although unable to provide 100% protection for EBIV-infected BALB/c mice, favipiravir has demonstrable antiviral efficiency against EBIV in vivo. The findings broaden the antiviral spectrum of favipiravir as an experimental countermeasure for orthobunyaviruses. Further studies are necessary for a better understanding of the exact mechanism of action of favipiravir against EBIV and to explore whether favipiravir can be used in synergy with other compounds.

## Data Availability

Data are contained within the article.

## Supplementary Materials

The following supporting information can be downloaded at: https://www.mdpi.com/article/10.3390/v16040631/s1, Figure S1: Body weight changes and viral copies on 1, 3, and 5 dpi of EBIV-infected BALB/c mice treated with vehicle or Favipiravir; Figure S2: Hematologic and and cytokine analysis on 1, 3, and 5 dpi in EBIV-infected mice with and without treatment; Figure S3: Blood chemistry analysis on 1, 3, and 5 dpi in EBIV-infected mice treated with favipiravir.
