# Supplementary material for: Favipiravir Treatment Prolongs Survival in a Lethal BALB/c Mouse Model of Ebinur Lake Virus Infection
Source: Viruses. 2024 Apr 18;16(4):631. doi: 10.3390/v16040631 (PMC11054260; doi:10.3390/v16040631)
Supplement: Supplementary file 1 [file viruses-16-00631-s001.zip › viruses-2937915-supplementary/Supplementary data.pdf]

Supplementary data

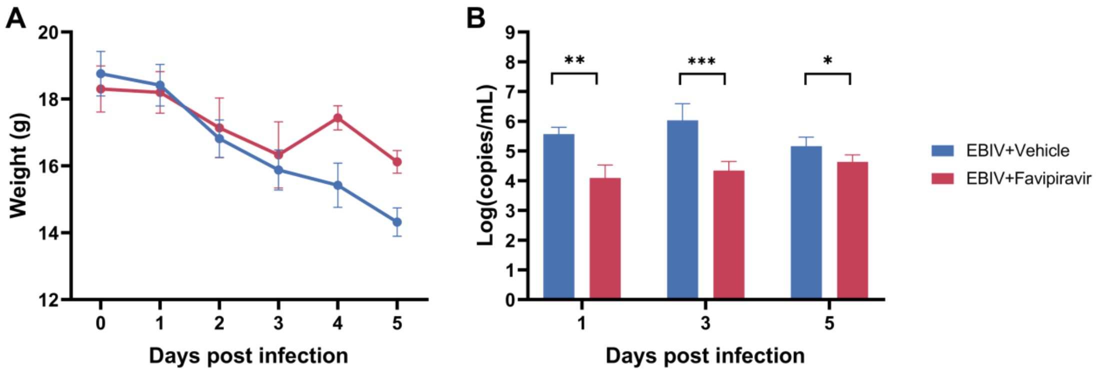

Figure S1. Body weight changes and viral copies on 1, 3, and 5 dpi of EBIV-infected BALB/c mice treated with vehicle or Favipiravir. Fifteen mice in each group were infected intraperitoneally with 10 PFU EBIV and treated intraperitoneally with vehicle or favipiravir. The treatment (administrated every 12 h) began at 2 days prior to EBIV infection and continued until 5 dpi. The weight data are represented as the group mean and standard error (A). The EBIV RNA load on 1, 3 and 5 dpi in the serum were determined by qRT-PCR (B). Asterisks denote significant differences (*: P<0.5, **: P<0.01 ***: *p* <0.001).

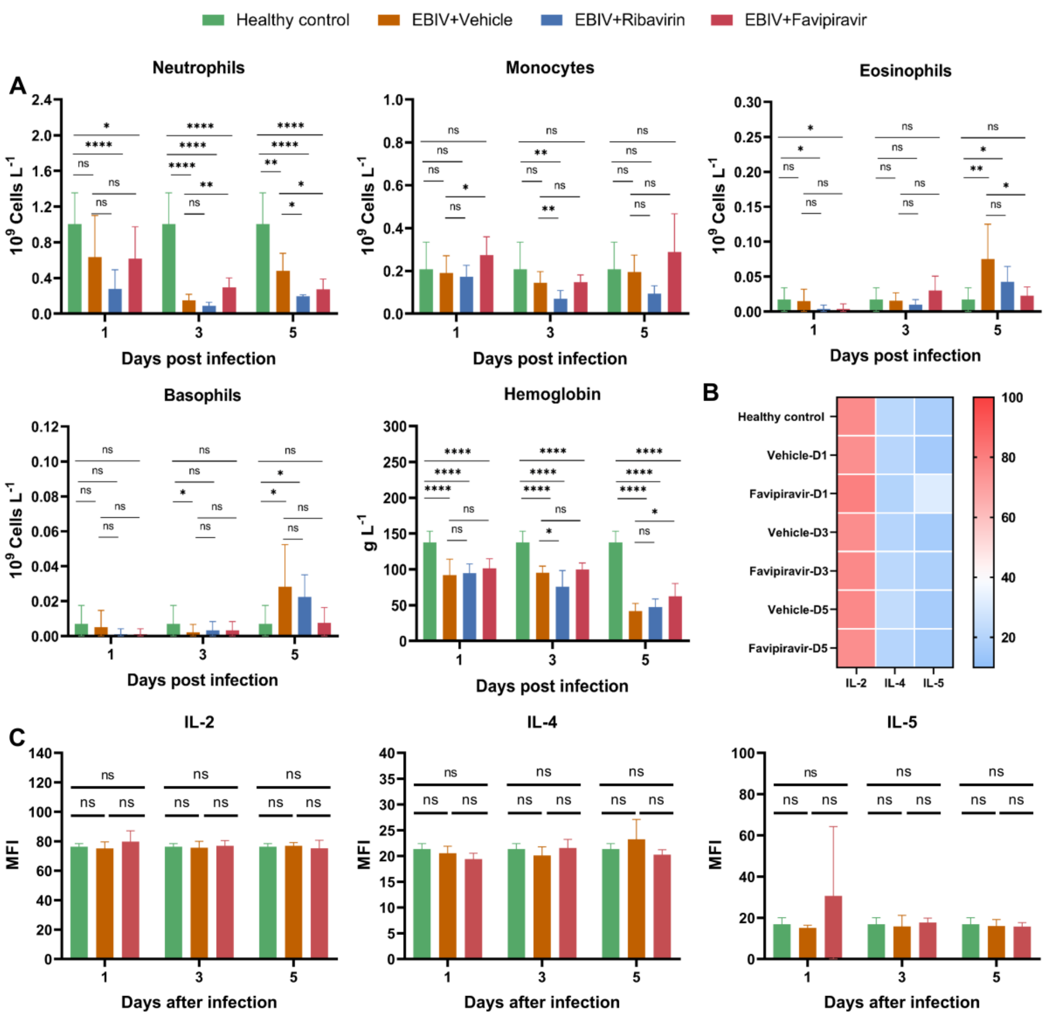

Figure S2. Hematologic and and cytokine analysis on 1, 3, and 5 dpi in EBIV-infected mice with and without treatment. Mice in each group were infected intraperitoneally with 10 PFU EBIV and treated intraperitoneally with vehicle, ribavirin or favipiravir. The treatment (administrated every 12 h) began at 2 days prior to virus infection and continued until the death or the body weight dropped by 25%. Healthy control showed the results using the blood samples collected from BALB/c mice without EBIV infection. The count of (A) Neutrophils; Monocytes; Eosinophils; Basophils and Hemoglobin (Hb); (B, C)The level of cytokines on 1, 3 and 5 dpi in EBIV infected BALB/c mice were shown. (*: p < 0.05, **: p <0.01, ***: p <0.001, ****: p <0.0001, ns: no significant difference).

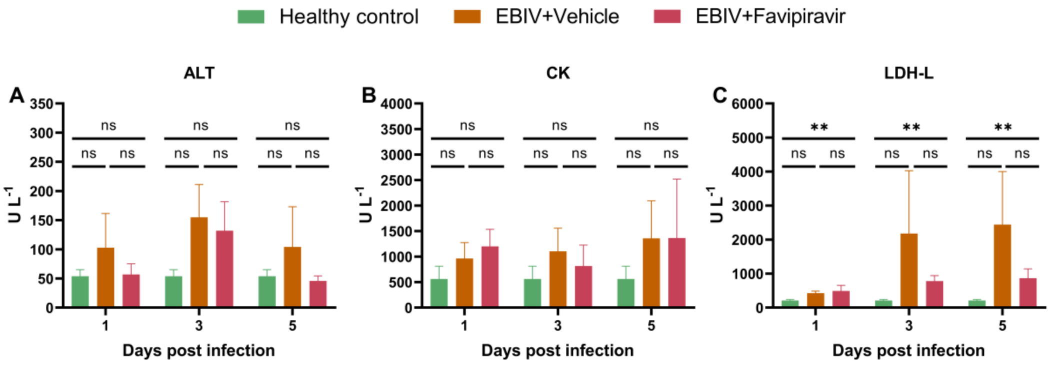

Figure S3. Blood chemistry analysis on 1, 3, and 5 dpi in EBIV-infected mice treated with favipiravir. Fifteen mice in each group were infected intraperitoneally with 10 PFU EBIV and treated intraperitoneally with vehicle or favipiravir. The treatment (administrated every 12 h) began at 2 days prior to virus infection and continued until 5 dpi. Five mice in each group were randomly sacrificed at 1, 3, and 5 dpi, respectively, and 5 healthy mice were also sacrificed to serve as healthy controls. (**: $p < 0.01$, ns: no significant difference).
